# Supplementary material for: Dermatopathological findings of Bothrops atrox snakebites: A case series in the Brazilian Amazon
Source: PLoS Negl Trop Dis. 2024 Dec 26;18(12):e0012704. doi: 10.1371/journal.pntd.0012704 (PMC11670982; doi:10.1371/journal.pntd.0012704)
Supplement: Attachment — Submitted filename: Reviewer-Resiere-PLOS 2024.pdf [file pntd.0012704.s001.pdf]

Reviewer comments:

The study on dermatopathological findings of *Bothrops atrox* snakebites in the Brazilian Amazon is a well-written and descriptive investigation. It is well-conducted and significantly contributes to the understanding and management of envenomation by the *Bothrops* genus of snake bites. The study describes the dermatopathological findings observed in a series of 22 patients admitted to a hospital in Manaus.

The study details both local and systemic tissue damage, including muscle necrosis and myonecrosis, which are characteristic of viperid snakebite envenomation. The tissue damage resulting from *Bothrops* envenomation is likely related to both direct venom activity and the inflammatory response or presence of an infectious process.

The severity grade of inflammatory infiltrate and fibrin was assessed. On histopathological evaluation, out of 22 patients, only 9 (40.9%) showed epidermal alterations. Spongiosis was observed in 3 cases (33.3%), with hyperkeratosis and acanthosis in 2 cases (22.2%) each (Figures 2, 4, and 5). Additionally, hyperplasia was found in 1 patient (11.1%), as well as hemorrhagic intraepidermal blister with neutrophil exocytosis (11.1%) and severe epidermis necrosis (11.1%).

A severe case of envenomation, whose biopsy was performed 2 days after the snakebite, presented with hemorrhage, inflammatory infiltrate, edema, congestion, vascular injury with thrombi formation, and collagen damage such as fiber lysis, hyaline necrosis, and skin appendage necrosis.

Minor Comments

1. The discussion could benefit from a comparison with similar studies conducted in other regions.
2. Why are there only a few cases of necrotizing fasciitis in your series?
3. In your series, you describe cases of severe manifestations of local and systemic envenomation. What was the appropriate treatment? Did these patients require specific treatments, several debridement procedures, and finally transfemoral amputation?

Conclusion

I do recommend minor revisions
