# Supplementary material for: Dermatopathological findings of Bothrops atrox snakebites: A case series in the Brazilian Amazon
Source: PLoS Negl Trop Dis. 2024 Dec 26;18(12):e0012704. doi: 10.1371/journal.pntd.0012704 (PMC11670982; doi:10.1371/journal.pntd.0012704)
Supplement: Attachment — Submitted filename: Response Letter_PLOS_NTD.docx [file pntd.0012704.s002.docx]

Dear Editor,

We appreciate you and the reviewers for your precious time in reviewing our paper and providing valuable comments. It was your valuable and insightful comments that led to possible improvements in the current version. The authors have carefully considered the comments and tried our best to address every one of them. We hope the manuscript after careful revisions meet your high standards. The authors welcome further constructive comments if any.

Below we provide the point-by-point responses. All modifications in the manuscript have been highlighted in yellow.

Sincerely,

Wuelton Monteiro, PhD

**Response to Reviewer 1**

**[General Comment]** The study on dermatopathological findings of Bothrops atrox snakebites in the Brazilian Amazon is a well-written and descriptive investigation. It is well-conducted and significantly contributes to the understanding and management of envenomation by the Bothrops genus of snake bites. The study describes the dermatopathological findings observed in a series of 22 patients admitted to a hospital in Manaus.

The study details both local and systemic tissue damage, including muscle necrosis and myonecrosis, which are characteristic of viperid snakebite envenomation. The tissue damage resulting from Bothrops envenomation is likely related to both direct venom activity and the inflammatory response or presence of an infectious process.

The severity grade of inflammatory infiltrate and fibrin was assessed. On histopathological evaluation, out of 22 patients, only 9 (40.9%) showed epidermal alterations. Spongiosis was observed in 3 cases (33.3%), with hyperkeratosis and acanthosis in 2 cases (22.2%) each (Figures 2, 4, and 5). Additionally, hyperplasia was found in 1 patient (11.1%), as well as hemorrhagic intraepidermal blister with neutrophil exocytosis (11.1%) and severe epidermis necrosis (11.1%).

A severe case of envenomation, whose biopsy was performed 2 days after the snakebite, presented with hemorrhage, inflammatory infiltrate, edema, congestion, vascular injury with thrombi formation, and collagen damage such as fiber lysis, hyaline necrosis, and skin appendage necrosis.

Some minor revisions for the authors to consider:

**[Minor Comment 1]:** The discussion could benefit from a comparison with similar studies conducted in other regions.

**Response**: Thank you for your suggestion. Dermatopathology results in human snakebite envenoming are still scarce but, currently, case reports describing these findings in viper and cobra envenoming have been arising and we were able to include them in the discussion of our work.

**[Minor Comment 2]:** Why are there only a few cases of necrotizing fasciitis in your series?

**Response**: The reviewer's question is very pertinent. According to our experience in the Brazilian Amazon, cases of necrotizing fasciitis following *Bothrops atrox* snakebites are very rare. None were seen during the study period.

**[Minor Comment 3]:** In your series, you describe cases of severe manifestations of local and systemic envenomation. What was the appropriate treatment?

**Response**: In our case series, bleeding disorders completely ceased within the first hours after antivenom treatment. All patients diagnosed with acute kidney injury were referred to the institution’s nephrologist. Those classified as stage III AKI underwent renal replacement therapy. Per current usual care, the affected limb was treated in the most comfortable position, according to patient preferences. When present, blisters were aspirated, necrotic tissue was surgically debrided, abscesses were drained, and antibiotic treatment was given accordingly.

**[Minor Comment 4]:** Did these patients require specific treatments, several debridement procedures, and finally transfemoral amputation?

**Response**: Per current usual care, the affected limb was treated in the most comfortable position, according to patient preferences. When present, blisters were aspirated, necrotic tissue was surgically debrided, abscesses were drained, and antibiotic treatment was given accordingly. There was no case of amputation of any kind.

**Response to Reviewer 2**

**[General Comment]** This study is a well-executed and methodologically sound piece of research that makes a significant contribution to the field. The authors have clearly articulated their objectives and have developed a testable hypothesis that is thoroughly supported by the data presented. The study design is robust, with an appropriate population and sample size that ensures sufficient statistical power. The analyses are consistent with the planned approach, and the results are presented with clarity and precision. The figures and tables are of high quality, further enhancing the communication of the findings.

The significance of the research is underscored by its relevance to public health, making it not only an academic contribution but also one with practical implications.

**Response**: Thank you very much for your previous comments that helped us improve this manuscript.

**Response to Reviewer 3**

**[General Comment]** In this article, the authors present 22 cases of *Bothrops atrox* envenomings and demonstrate their clinicopathological observations. While this is an important topic and very little is known about this issue, the manuscript needs a significant revision to add more to the existing case report-based literature.

**Response**: Thank you very much for your comments that helped us improve this manuscript.

Some major revisions for the authors to consider:

**[Major Comment 1]:** I would strongly suggest the authors thoroughly proofread this manuscript as there are numerous typographical and grammatical errors throughout the article.

**Response:** Thanks for your suggestion. A native English speaker revised the manuscript.

**[Major Comment 2]:** Please separate the figure and table legends from the main text and present them separately.

**Response:** Done accordingly.

**[Major Comment 3]:** It looks like this is not the final version of the manuscript as it has several track changes. So please make sure to submit the final version of the manuscript that has been approved by all the authors.

**Response:** Thank you very much for the reminder. Done accordingly.

**[Major Comment 4]:** The authors have collected a lot of data from these patients, so they need to perform some form of analysis to compare and contrast the cases or groups. For example, the cases in mild, moderate and severe oedema categories can be grouped, and compared and contrasted with the dermopathological findings with other parameters. In which patients do CK and LDH increase and why? Link the data to inappropriate first aid used. Compare the data between males and females. compare between different ages or times of arrival to the hospital?

**Response:** Thank you very much for your suggestions. Although the authors understand the importance of statistical analysis to draw conclusions on association or causation, the design of our study is a case series, with descriptive results from a limited number of cases. *The stratification of the 22 patients included in this study into subgroups for comparison did not allow us to observe any significant difference in histopathological findings according to sex, age, severity classification, time to medical assistance, time from bite to biopsy, first aid used, and clinical and laboratorial variables (CK and LDH increase, for instance).* ***This was the number of patients who agreed to participate in the study and undergo biopsy.***

These observations do not necessarily mean that dematopathological alterations are not dependent on the characteristics mentioned above, but they may reflect a type II error likely to occur when sample sizes are too small. Although case series design is not considered the strongest source of evidence for clinical decisions, those are particularly important to provide descriptive novelty and contributes to building knowledge and generating hypotheses (1,2).

References:

Vandenbroucke JP. In defense of case reports and case series. Ann Intern Med. 2001 Feb 20;134(4):330-4.

Murad MH, Sultan S, Haffar S, Bazerbachi F. Methodological quality and synthesis of case series and case reports. BMJ Evid Based Med. 2018;23(2):60-63.

**[Major Comment 5]:** Similarly, various parameters can be quantified using the micrographs of skin, and they can be compared between different cohorts to draw firm conclusions.

**Response:** Please see Response to Major Comment 4.

**[Major Comment 6]:** Please show data for CK and LDH in graphs for different groups.

**Response:** Please see Response to Major Comment 4.

**[Major Comment 7]:** The results section should be expanded by elaborating on the data by discussing various aspects. Otherwise, this will simply look like another case report but with 22 patients.

**Response:** Please see Response to Major Comment 4.

**[Major Comment 8]:** The discussion section is unusually long compared to the small results section (without table and figure legends). So please cut down the unnecessary parts of the discussion and keep the focus only on the local envenomation effects, and how they can be linked to mechanistic details as well as inappropriate and appropriate timely treatment.

**Response:** Thank you very much for your suggestion. We have made revisions accordingly.
