# Supplementary material for: Dermatopathological findings of Bothrops atrox snakebites: A case series in the Brazilian Amazon
Source: PLoS Negl Trop Dis. 2024 Dec 26;18(12):e0012704. doi: 10.1371/journal.pntd.0012704 (PMC11670982; doi:10.1371/journal.pntd.0012704)
Supplement: Attachment — Submitted filename: 2. Response Letter_PLOS_NTD.docx [file pntd.0012704.s003.docx]

Dear Editor,

We appreciate you and the reviewers for your precious time in reviewing our paper and providing valuable comments. It was your valuable and insightful comments that led to possible improvements in the current version. The authors have carefully considered the comments and tried our best to address every one of them. We hope the manuscript after careful revisions meet your high standards. The authors welcome further constructive comments if any.

Below we provide the point-by-point responses. All modifications in the manuscript have been highlighted in yellow.

Sincerely,

Wuelton Monteiro, PhD

**Additional Editor Comments (if provided):**

The reviewers appreciat the modifications introduced to this manuscript. One reviewer, however, raised some minor issues that should be considered for preparing a new revised version.

**Response**: Thank you very much for your comments that helped us improve this manuscript.

**Methods**

Reviewer #1: The objectives are well defined with a population clearly described.

Reviewer #2: The objectives is clearly articulated
The design of the study is appropriated
The population is clearly described
The sample size is small however the design the study is a case series with a descriptive results from a limited number of cases

Reviewer #3: (No Response)

**Response**: Thank you very much for your comments that helped us improve this manuscript.

**Results**

-Does the analysis presented match the analysis plan?
-Are the results clearly and completely presented?
-Are the figures (Tables, Images) of sufficient quality for clarity?

Reviewer #1: The results are completely presented

Reviewer #2: Yes, the analysis aligns well with the analysis plan, the results are presented clearly and comprehensively, and the figures (tables and images) are of high quality, ensuring clarity.

Reviewer #3: (No Response)

**Response**: Thank you very much for your comments that helped us improve this manuscript.

**Conclusions**

-Are the conclusions supported by the data presented?
-Are the limitations of analysis clearly described?
-Do the authors discuss how these data can be helpful to advance our understanding of the topic under study?
-Is public health relevance addressed?

Reviewer #1: The conclusions supported the presented datas. This is a public health issue

Reviewer #2: The conclusions support the data presented, the limitations are clearly described, the advance of the study is discussed. The public health relevance was addressed.

Reviewer #3: (No Response)

**Response**: Thank you very much for your comments that helped us improve this manuscript.

**Editorial and Data Presentation Modifications?**

Use this section for editorial suggestions as well as relatively minor modifications of existing data that would enhance clarity. If the only modifications needed are minor and/or editorial, you may wish to recommend “Minor Revision” or “Accept”.

Reviewer #1: Thuis paper needs minor modifications

Reviewer #2: No modifications is required.

Reviewer #3: (No Response)

**Response**: Thank you very much for your comments that helped us improve this manuscript.

**Summary and General Comments**

Use this section to provide overall comments, discuss strengths/weaknesses of the study, novelty, significance, general execution and scholarship. You may also include additional comments for the author, including concerns about dual publication, research ethics, or publication ethics. If requesting major revision, please articulate the new experiments that are needed.

Reviewer #1: Reviewer comments:
The study on dermatopathological findings of Bothrops atrox snakebites in the Brazilian Amazon is a well-written and descriptive investigation. It is well-conducted and significantly contributes to the understanding and management of envenomation by the Bothrops genus of snake bites. The study describes the dermatopathological findings observed in a series of 22 patients admitted to a hospital in Manaus. The study details both local and systemic tissue damage, including muscle necrosis and myonecrosis, which are characteristic of viperid snakebite envenomation. The tissue damage resulting from Bothrops envenomation is likely related to both direct venom activity and the inflammatory response or presence of an infectious process. The severity grade of inflammatory infiltrate and fibrin was assessed. On histopathological evaluation, out of 22 patients, only 9 (40.9%) showed epidermal alterations. Spongiosis was observed in 3 cases (33.3%), with hyperkeratosis and acanthosis in 2 cases (22.2%) each (Figures 2, 4, and 5). Additionally, hyperplasia was found in 1 patient (11.1%), as well as hemorrhagic intraepidermal blister with neutrophil exocytosis (11.1%) and severe epidermis necrosis (11.1%). A severe case of envenomation, whose biopsy was performed 2 days after the snakebite, presented with hemorrhage, inflammatory infiltrate, edema, congestion, vascular injury with thrombi formation, and collagen damage such as fiber lysis, hyaline necrosis, and skin appendage necrosis.
Minor Comments
1. The discussion could benefit from a comparison with similar studies conducted in other regions.

**Response**: Thank you for your suggestion. Dermatopathology studies in human snakebites are still rare and were included in the discussion of our work. However, we emphasize that almost all information in the literature on this topic comes from animal models.

2. Why are there only a few cases of necrotizing fasciitis in your series?

**Response**: The reviewer's question is very pertinent. According to our experience in the Brazilian Amazon, cases of necrotizing fasciitis following *Bothrops atrox* snakebites are very rare. None were seen during the study period.

We believe that this fact is due to the absence or rarity of beta-hemolytic *Streptococcus*, the main cause of necrotizing fasciitis [1,2], in the oral microbiota of snakes of the genus *Bothrops* [3].

References:

1. Guliyeva G, Huayllani MT, Sharma NT, Janis JE. Practical Review of Necrotizing Fasciitis: Principles and Evidence-based Management. Plast Reconstr Surg Glob Open. 2024 Jan 19;12(1):e5533.
2. Allaw F, Wehbe S, Kanj SS. Necrotizing fasciitis: an update on epidemiology, diagnostic methods, and treatment. Curr Opin Infect Dis. 2024 Apr 1;37(2):105-111.
3. Larréché S, Bousquet A, da Silva L, Planelles A, Ksas R, Mérens A, Chippaux JP. Antibiotic susceptibility of cultivable microbiota from the oral cavity of captive *Bothrops atrox* and *Bothrops lanceolatus*: Implications for the treatment of snakebite-associated infections in the French departments of America. Infect Dis Now. 2023 Oct;53(7):104721.

3. In your series, you describe cases of severe manifestations of local and systemic envenomation. What was the appropriate treatment? Did these patients require specific treatments, several debridement procedures, and finally transfemoral amputation?
**Response**: In our case series, bleeding disorders completely ceased within the first hours after antivenom treatment. All patients diagnosed with acute kidney injury were referred to the institution’s nephrologist. Those classified as stage III AKI underwent renal replacement therapy. Per current usual care, the affected limb was treated in the most comfortable position, according to patient preferences. When present, blisters were aspirated, necrotic tissue was surgically debrided, abscesses were drained, and antibiotic treatment was given accordingly.

Per current usual care, the affected limb was treated in the most comfortable position, according to patient preferences. When present, blisters were aspirated, necrotic tissue was surgically debrided, abscesses were drained, and antibiotic treatment was given accordingly. There was no case of amputation of any kind.

Reviewer #2: This study is methodologically robust and well-conducted, offering a valuable contribution to the field. The authors clearly outline their objectives and present a testable hypothesis, which is well-supported by the data. The study design is solid, featuring an appropriate population and sample size that provide adequate statistical power. The analyses align with the planned methodology, and the results are conveyed with clarity and accuracy. High-quality figures and tables further improve the presentation of the findings.

**Response**: Thank you very much for your comments that helped us improve this manuscript.

Reviewer #3: (No Response)
